# Investigations of Electron-Electron and Interlayer Electron-Phonon Coupling in van der Waals hBN/WSe_2_/hBN Heterostructures by Photoluminescence Excitation Experiments

**DOI:** 10.3390/ma14020399

**Published:** 2021-01-15

**Authors:** Joanna Jadczak, Joanna Kutrowska-Girzycka, Janina J. Schindler, Joerg Debus, Kenji Watanabe, Takashi Taniguchi, Ching-Hwa Ho, Leszek Bryja

**Affiliations:** 1 Department of Experimental Physics, Wrocław University of Science and Technology, Wybrzeże Wyspiańskiego 27, 50-370 Wroclaw, Poland; joanna.kutrowska-girzycka@pwr.edu.pl; 2 Experimentelle Physik 2, Technische Universität Dortmund, 44227 Dortmund, Germany; janina.schindler@tu-dortmund.de (J.J.S.); joerg.debus@tu-dortmund.de (J.D.); 3 National Institute for Materials Science, Tsukuba 305-0044, Ibaraki, Japan; WATANABE.Kenji.AML@nims.go.jp (K.W.); taniguchi.takashi@nims.go.jp (T.T.); 4 Graduate Institute of Applied Science and Technology, National Taiwan University of Science and Technology, Taipei 106, Taiwan; chho@mail.ntust.edu.tw

**Keywords:** transition metal dichalcogenides monolayer, van der Waals heterostructures, photoluminescence excitation, reflectivity, Raman scattering

## Abstract

Monolayers of transition metal dichalcogenides (TMDs) with their unique physical properties are very promising for future applications in novel electronic devices. In TMDs monolayers, strong and opposite spin splittings of the energy gaps at the K points allow for exciting carriers with various combinations of valley and spin indices using circularly polarized light, which can further be used in spintronics and valleytronics. The physical properties of van der Waals heterostructures composed of TMDs monolayers and hexagonal boron nitride (hBN) layers significantly depend on different kinds of interactions. Here, we report on observing both a strong increase in the emission intensity as well as a preservation of the helicity of the excitation light in the emission from hBN/WSe_2_/hBN heterostructures related to interlayer electron-phonon coupling. In combined low-temperature (*T* = 7 K) reflectivity contrast and photoluminescence excitation experiments, we find that the increase in the emission intensity is attributed to a double resonance, where the laser excitation and the combined Raman mode A′_1_ (WSe_2_) + ZO (hBN) are in resonance with the excited (2s) and ground (1s) states of the A exciton in a WSe_2_ monolayer. In reference to the 2s state, our interpretation is in contrast with previous reports, in which this state has been attributed to the hybrid exciton state existing only in the hBN-encapsulated WSe_2_ monolayer. Moreover, we observe that the electron-phonon coupling also enhances the helicity preservation of the exciting light in the emission of all observed excitonic complexes. The highest helicity preservation of more than 60% is obtained in the emission of the neutral biexciton and negatively charged exciton (trion) in its triplet state. Additionally, to the best of our knowledge, the strongly intensified emission of the neutral biexciton XX^0^ at double resonance condition is observed for the first time.

## 1. Introduction

Atomically thin two dimensional (2D) crystals with their unique physical properties have attracted considerable attention as a new platform for scientific and technological studies [1,2,3,4,5,6,7,8,9,10,11,12,13,14,15,16,17,18,19,20]. Monolayers of group-VI transition metal dichalcogenides (TMDs), such as MoS_2_ and WSe_2_, with a direct band gap in the visible energy range, have emerged as an alternative for the zero-energy-gap monolayer graphene [21,22,23]. Like in graphene, the energy gap of TMDs monolayers is positioned at inequivalent K^+^ and K^−^ points of the 2D hexagonal Brillouin zone. A unique characteristic of group-VI TMDs monolayers is spin-valley locking [4,5,6,7]. The lack in inversion symmetry and the presence of strong spin-orbit coupling in a monolayer system result in valley-contrasting and strong spin splittings of the valence and conduction bands at the K^+^ and K^−^ valleys. They allow for exciting carriers with various combinations of valley and spin indices using circularly polarized light [4,5,6,7]. Due to weak van der Waals bonds between the atomic layers in graphene, TMDs, hexagonal boron nitride (hBN) and other 2D crystals, they can easily be assembled into so called van der Waals heterostructures which are made by atomic layers stacked in a chosen sequence [24]. The layer-layer interaction gives rise to a variety of unusual physical effects in such heterostructures. The interlayer electron-electron interactions lead to effects like, for example, moiré excitons in MoSe_2_/MoSe_2_ heterostructures [25,26,27] and cloning of Dirac fermions in graphene superlattices [28,29,30]. In addition to interlayer electron-electron interactions, the interlayer electron-phonon coupling has a strong impact on the physical properties of the van der Waals heterostructures and can improve or limit the performance of devices based on those structures. For example, the coupling between electrons in FeSe films with phonons in a SrTiO_3_ substrate leads to a significant enhancement of the critical superconductivity temperature [31,32]. On the other hand, the interlayer electron-phonon interaction considerably limits the mobility of transistors based on graphene monolayers exfoliated directly on SiO_2_/Si substrates [33]. However, the exfoliation of graphene monolayers on smooth high-purity hBN crystals allows for elaborating devices with ultrahigh mobility [24,34]. As for graphene, the encapsulation of transition metal dichalcogenides monolayers within high-purity hBN flakes significantly improves the monolayer quality resulting in a substantial narrowing of the emission lines. Accordingly, rich emission spectra with coexisting neutral and charged excitons and biexcitons are observed [35,36,37]. In addition to that, the hBN-based encapsulation of TMDs monolayers remarkably affects the efficiency of the monolayer emission which was demonstrated in recent studies of WSe_2_ and MoSe_2_ van der Waals heterostructures [38,39,40].

Here, we report on combined low-temperature (*T* = 7 K) reflectivity contrast (RC) and photoluminescence excitation (PLE) experiments of a WSe_2_ monolayer encapsulated in hBN crystals of superior quality. We observe a strong intensity enhancement of the emission from the WSe_2_ monolayer due to interlayer electron-phonon coupling. The highest increase of the emission intensity is related to a double resonance, where the laser excitation is in resonance with the 2s A exciton of the WSe_2_ monolayer and the energy of the combined Raman scattering mode A′_1_ (WSe_2_) + ZO (hBN) is equal to the energy separation between the 2s and 1s exciton states. This assignment is in contrast to a recent paper [38] which claims that the outgoing photon energy corresponds to the energy of the 1s A exciton in the WSe_2_ monolayer and the ingoing photon is in resonance with a not further elaborated hybrid state existing only in hBN/WSe_2_/hBN. The Raman mode observed in the resonance process corresponds to the superposition of two phonon modes: The A′_1_ mode from WSe_2_ and the ZO mode from hBN. The latter mode is silent in pure hBN and becomes strongly intensified in hBN/WSe_2_/hBN heterostructures due to its resonant coupling with WSe_2_ electronic transitions. We further reveal a pronounced effect of layer-layer interactions on the circular polarization degree of the WSe_2_ emission. Due to the electron-phonon coupling the helicity of the exciting light is preserved in the emission of all observed excitonic complexes, for the neutral biexciton and negative triplet trion the helicity preservation reaches about 60%. In comparison to that, the maximum helicity preservation is about two times smaller for the neutral A exciton. To the best of our knowledge, the intensity of the neutral biexciton emission enhanced remarkably at the double resonance condition is observed for the first time.

## 2. Materials and Methods

The WSe_2_ monolayers studied here were prepared by mechanical exfoliation of bulk crystals grown by the chemical vapour transport (CVT) technique. Prior to the crystal growth, a powdered compound was prepared from the elements (W: 99.99%; Se: 99.999%) by chemical reaction at *T* = 1000 °C for 10 days in evacuated quartz ampoules. The slow heating (from Lindberg Blue M three-zone tube furnace, RT to 1100 °C) was necessary to avoid any explosions due to the strongly exothermic reaction between the elements. The chemical transport was achieved with Br_2_ as a transport agent having a density of about 5 mg/cm^3^. The growth temperature was set from 1030 to 980 °C, with a temperature gradient of 3 °C/cm. In order to grow large single crystals with a high thickness, the growth of the WSe_2_ crystals lasted for 20 days. The longer growth time provided enough period for seed-nucleation and crystal formation being progressed in the growth. The crystals had the shape of thin layered plates with thicknesses and surface areas ranging from 30 to 1000 µm and 50 to 100 mm^2^, respectively. X-ray diffraction measurements (Bruker D2 Phaser X-ray diffractometer) confirmed that the crystal stacking had a two-layer hexagonal (2H) structure [41].

**Figure 1:**
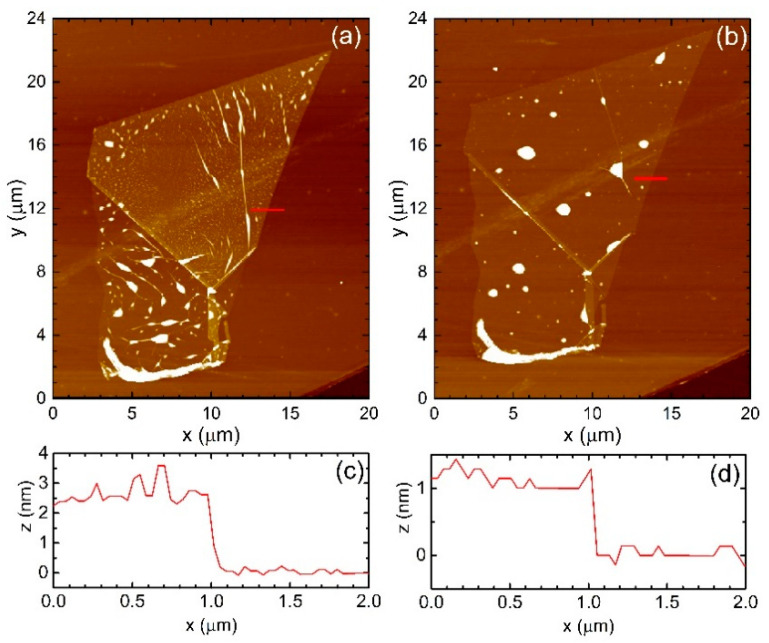
AFM images of the WSe_2_ flake on the top of the annealed hexagonal boron nitride (hBN) layer: (**a**) before and (**b**) after the annealing for 20 min at 180 °C in air. Line profiles of the red lines shown in the AFM images (**c**) before and (**d**) after annealing.

We prepared van der Waals hBN/WSe_2_/hBN/SiO_2_/Si heterostructures using high-purity hBN [42]. The WSe_2_ monolayers and the hBN flakes with different thicknesses were mechanically exfoliated and then stacked using the deterministic all-dry stamping method on Si substrates with 300 nm SiO_2_. Typical flake sizes exceeded 10 µm × 10 µm. To improve the contact between the transferred layers, immediately after the transfer of each subsequent layer, the sample was annealed for 20 min at a temperature of 180 °C on a hot plate in air. Additionally, after the transfer of the last top hBN layer the heterostructure was annealed for 2 h at 200 °C in air. Figure 1a–d present atomic force microscopy (AFM) images (by Park Systems XE7, Park Systems KANC 4F, Suwon, South Korea) and line profiles of the WSe_2_ flake on the top of the annealed hBN layer before and after the annealing, respectively. It is visible that strain-induced bubbles and wrinkles are reduced after the annealing and aggregates in some areas of the WSe_2_ flake. The comparison of the line profiles shows an improvement of the contact between the layers after annealing. The monolayer character of the WSe_2_ flakes was determined by their different optical contrast and was confirmed by Raman scattering and photoluminescence (PL) measurements on polydimethylsiloxane at the same experimental conditions. The Raman and PL spectra are collected using a Renishaw inVia confocal Raman microscope (Renishaw plc, Gloucestershire, UK).

The samples were mounted on the cold finger of a non-vibrating closed-cycle helium cryostat (model DE-204AF, Advanced Research Systems, Inc., Macungie, PA, USA), where the temperature could be varied from 7 to 350 K. PL was excited either by the second harmonic 532 nm (2.33 eV) of a continuous-wave single-mode Nd:YAG laser or by a continuous-wave dye laser equipped with DCM and tunable in the range from 610 nm to 685 nm (from 2.03 eV to 1.81 eV, respectively). The laser beam was focused on the sample under normal incidence using a high resolution, long-working distance (WD = 10 mm, NA = 0.65) 50× microscope objective (Plan Apo NIR HR Infinity-Corrected Objective, Mitutoyo, Sakado, Japan). The diameter of the excitation spot was about 1 µm. The emission from the sample was collected by the same microscope objective and was analyzed with a 0.5-m-focal length spectrometer (Acton SP2500, Princeton Instruments, Acton, MA, USA) equipped with a 600 lines/mm grating and a Peltier-cooled charged coupled device Si camera (Excelon BR 1024 × 256 pixels, Princeton Instruments, Trenton, NJ, USA). The RC spectra were measured at the same setup using a filament lamp as light source (HL-2000, Ocean Optics Asia, Shanghai, China). The Raman scattering spectra were obtained in the backscattering geometry. The polarization of the excitation and detection light was set each by a linear polarizer combined with a λ/2 or λ/4 retardation plate. To reduce scattered laser light, appropriate long- and short-pass edge filters were used. 

## 3. Results and Discussion

**Figure 2:**
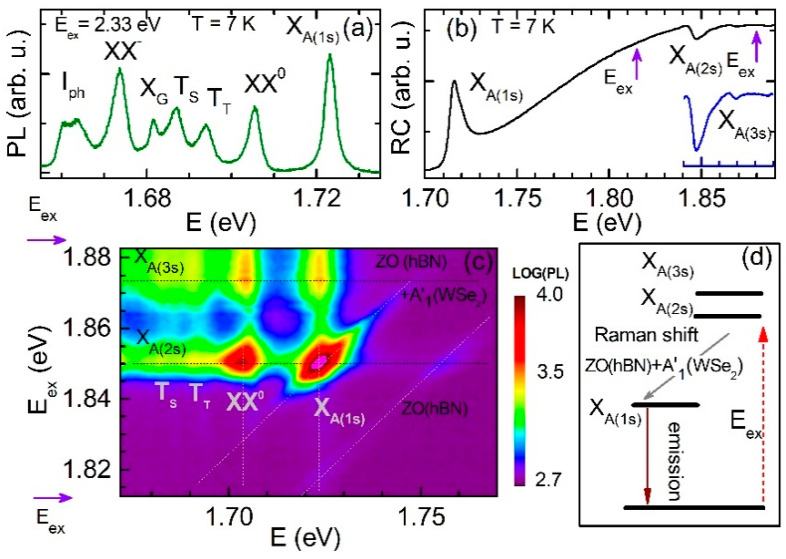
Unpolarized photoluminescence and reflectivity contrast spectra of hBN/WSe_2_/hBN heterostructure recorded at *T* = 7 K. (**a**) PL spectrum excited non-resonantly with laser energy E_ex_ = 2.33 eV. (**b**) RC spectrum recorded at the spectral range of the A exciton transitions: 1s, 2s and 3s. Violet arrows mark the range of energy of laser excitation applied in the photoluminescence excitation experiment. (**c**) PLE spectra recorded at the energy range marked in (**b**) by violet arrows. (**d**) The schematic representation of the double resonance process.

Due to the two-dimensional confinement and reduced dielectric screening, the optical spectra of monolayer TMDs are dominated by excitonic transitions as in the case of other 2D semiconductor heterostructures [43,44,45]. Figure 2a presents a PL spectrum measured at *T* = 7 K and non-resonant 2.33 eV laser excitation. The spectrum contains several emission lines exhibiting characteristics which were reported for WSe_2_ monolayers encapsulated in high-quality hBN flakes [35,36,37]. The highest energy emission peak detected at E = 1.725 eV is assigned to the neutral A exciton (X_A_) in the ground state (1s). For a WSe_2_ monolayer, the optically active A exciton is associated with the top spin-split valence subband and the upper spin-split conduction subband [46]. The exciton associated with the top spin-split valence subband and the lower spin-split conduction subband is optically inactive; accordingly, it is called a dark exciton [46]. The B exciton is formed by the heavy hole at the lower spin-split valence band and the electron at the lower spin-split conduction band. It is detected in optical spectra at high energy of about 2.2 eV [47,48] due to the large spin splitting of the valence band Δ_vb_ which is equal to about 0.5 eV [46]. The spin splitting of the conduction band Δ_cb_ is much smaller and equals to ~30 meV [49]. The peak positioned at the energy E = 1.710 eV is attributed to the neutral biexciton (XX^0^) resonance. The two peaks located at E = 1.695 eV and E = 1.685 eV stem from the radiative recombination of the negatively charged excitons (trions), namely the intravalley spin-triplet and intervalley spin-singlet trions (T_T_ and T_S_, respectively). The peak detected at E = 1.681 eV is attributed to the resonance of the spin-forbidden dark exciton; it is called gray exciton (X_G_). The emission of bright excitons (of, e.g., X_A_ or XX^0^) propagates perpendicular to the monolayer plane due to their in-plane optical dipole momenta. The dark exciton emission propagates along the WSe_2_ monolayer plane, since it interacts with light by the spin-flip dipole matrix element whose direction is perpendicular to the monolayer plane (out-of-plane). The emission of X_G_ is recorded in the PL spectra, since a high-numerical aperture objective (NA = 0.65) was used which collected a part of the out-of-plane polarized emission [37,50]. The PL spectrum shown in Figure 2a is completed by a peak at E = 1.673 eV corresponding to the negatively charged biexciton (XX^−^) and by a pair of zone-corner chiral-phonon replicas of the dark exciton states (I_ph_) detected at E = 1.665 eV and E = 1.661eV [51].

In order to get further inside into the exciton properties, we performed reflectivity contrast experiments at *T* = 7 K in the energy range near and above the energy of the A exciton in its ground state. Figure 2b displays the RC spectrum which is defined as ΔR/R =(R_mon_ − R_sub_)/R_sub_, where R_mon_ and R_sub_ indicates the reflectivity recorded from the monolayer and substrate, respectively. At the high-energy side of the excitonic ground state, two additional resonances appear as dips in the spectrum. We attribute them to the excited exciton states 2s and 3s. We use the notation of an excitonic Rydberg series in analogy to the hydrogen atom [52]. Our interpretation is based on the observation that both the energy separation as well as the amplitude of these resonances decrease with increasing energy following the pattern characteristic for the excitonic Rydberg series [52]. From the comparison of the PL and RC spectra, we see that the maximum PL intensity of the 1s A exciton coincides energetically with the lowest energy dip in the RC spectrum. This observation allows us to determine the energies of the excited states, 2s and 3s, of the A exciton by the subsequent dips in the RC spectrum positioned at the energies E = 1.848 eV and E = 1.875 eV, respectively. The ground and excited states from 2s up to 5s of the A exciton were observed in a previous study of a WSe_2_ monolayer using linear absorption and two-photon PLE spectroscopy [9]. The energies of the A exciton states in ref. [9] slightly differ from our results. This is related to different dielectric environments of the WSe_2_ monolayer in both experiments which significantly affects the screening of the Coulomb electron-hole interaction. The shifts in the emission and absorption excitonic resonances governed by the different dielectric environments of the TMDs monolayers were observed in previous works [15,17]. Resonances from negative trions are not found in the RC spectra. This fact along with the appearance of the multiple A exciton states indicates that the two-dimensional electron gas (2DEG) concentration in our WSe_2_ monolayer, related to unintentional residual doping, is low but still significant, since we obtain charged excitons and charged biexcitons in the PL. 

Next, we demonstrate PLE results for tuning the laser excitation energy from E_ex_ = 1.88 eV (above the energy of the A exciton in the 3s state) down to E_ex_ = 1.81 eV. Figure 2c shows a 2D color plot which combines the PLE spectra measured. The horizontal and vertical axes correspond to the emission and excitation energy, respectively. The emission intensity is displayed in the color logarithmic scale. In comparison to the non-resonantly excited PL (see Figure 2a), two additional peaks appear at the high energy side of the emission spectrum. They shift linearly with changes in the laser excitation energy. The peaks were reported in previous studies and were attributed to Raman scattering phonon modes [38,39]. The mode observed at higher energy was attributed to the ZO hBN mode which is silent in Raman scattering spectra of an isolated hBN. However, it gains intensity by more than two orders of magnitude due to the resonant coupling to electronic transitions in the WSe_2_ monolayer. The mode at lower energy is a superposition of the ZO mode from the hBN layer and the A′_1_ mode from the WSe_2_ monolayer. When the combined mode ZO (hBN) + A′_1_(WSe_2_) matches energetically with the radiative recombination of the A exciton (X_A_), its emission and that of the biexciton (XX^0^) become strongly increased. The enhanced emission of the neutral exciton was also found in a previous study of the similar heterostructure performed at a higher temperature of *T* = 77 K [38]. It was attributed to a double resonance in which the outgoing photon energy corresponds to the energy of the 1s A exciton in the WSe_2_ monolayer, whereas the ingoing laser excitation was in resonance with a hybrid state existing only in hBN/WSe_2_/hBN. However, as one can clearly evaluate from the RC and PLE spectra recorded for our heterostructure (Figure 2b,c, respectively) the outgoing (ingoing) photon is in resonance with the 1s (2s) A exciton. This double resonance process is schematically presented in Figure 2d. Our observation that in the double resonance process two states of the same exciton (1s and 2s) are involved allows for a comprehensive explanation of the considerably enhanced emission intensity. Furthermore, to the best of our knowledge the strong increase in the emission intensity of the neutral biexciton XX^0^ at the double resonance condition is observed for the first time. It is likely due to the fact that the XX^0^ state is a combination of the bright (spin-allowed) and long-lived dark (spin-forbidden) excitons residing at the K and K′ valleys, respectively [36,53]. Interestingly, the intensities of all detected PL lines are enhanced when the laser excitation energy is equal to the energy of the 3s A exciton state at 1.875 eV. In this case the intensity enhancement is not as strong as in the double resonance process, since the former process is governed by a single resonance when only the ingoing photon resonantly excites the 2s A exciton.

To get further inside into the nature of the electron-phonon coupling in the hBN-encapsulated WSe_2_ monolayer, we study the dependence of the circular polarization degree of the PL lines on the helicity of the laser light as a function of the excitation energy. The circular polarization degrees of the excitonic complexes in different TMDs monolayers have been studied and discussed in detail in previous works [15,54,55]. There is a common agreement that the most efficient mechanism responsible for quenching the circular polarization of the excitonic emission is the scattering of neutral excitons X_A_ between the different K^+^ and K^−^ valleys. Other relaxation processes, such as scattering of singlet and triplet trions between the K^+^ and K^−^ valleys, are likely less efficient, since they require spin flips of the electrons and holes.

**Figure 3:**
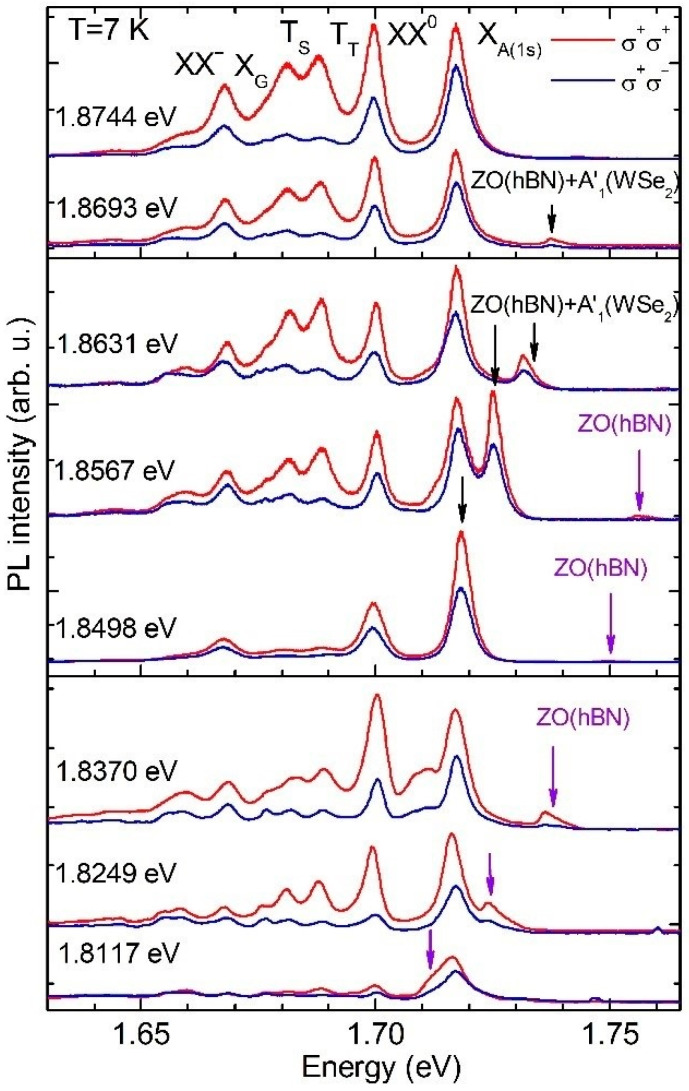
Helicity-resolved PL spectra recorded in two circular polarization configurations of excited and emitted light: The same (co—polarized, σ^+^/σ^+^—red line) and opposite (cross-polarized, σ^+^/σ^−^—blue line) polarizations. Excitation energies are marked at the left corner of each spectrum. The energy positions of PL lines are marked in the upper panel. The Raman phonon modes ZO (hBN) + A′_1_(WSe_2_) and ZO (hBN) are marked by black and violet arrows, respectively.

**Figure 4:**
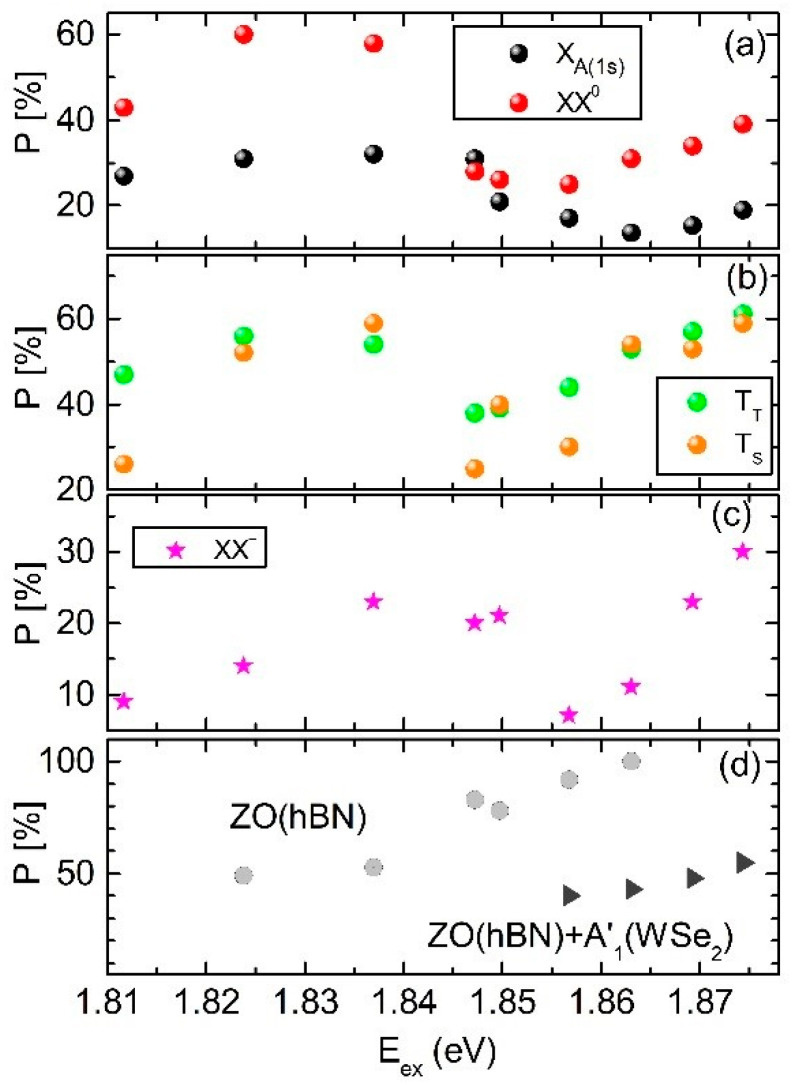
Degree of circular polarization plotted as a function of excitation energy for: (**a**) neutral A exciton (X_A_) and biexciton (XX^0^), (**b**) negative trion in the singlet (T_S_) and triplet (T_T_) state, (**c**) negatively charged biexciton (XX^−^), (**d**) Raman modes ZO (hBN) and ZO (hBN) + A′_1_ (WSe_2_).

In our study, we address the impact of the interlayer electron-phonon coupling on the preservation of the exciting light helicity in the emission from the hBN/WSe_2_/hBN heterostructure. Figure 3 shows the helicity-resolved emission spectra of the studied structure detected at different excitation energies ranging from E_ex_ = 1.8774 eV to E_ex_ = 1.8117 eV. This variation corresponds to the energy range applied in the unpolarized PLE experiments. The spectra were recorded in two circular polarization configurations of the exciting and emitted light: co–polarized, σ^+^/σ^+^ (red line) and cross-polarized, σ^+^/σ^−^ (blue line). Figure 4 shows the respective evolution of the circular polarization degree of each individual PL line. It is defined by P = (I_co_ − I_cross_)/(I_co_ + I_cross_), where I_co_ and I_cross_ indicate the PL intensity in the co-polarized and cross-polarized configuration, respectively. The PL intensities were determined by fitting procedures in which each line was modeled by a Gaussian curve. As seen from Figure 3 and Figure 4, each PL line exhibits its own behavior. However, some common features are figured out in the evolution of the different polarization degrees. 

The polarization degrees of all lines have a local maximum when the laser excitation energy E_ex_ matches with the energy of the A exciton in its excited state 3s at 1.8744 eV. When E_ex_ is lowered, the polarization degrees of all lines decrease at first and then they gradually increase when the excitation energy crosses the energy corresponding to the double resonance observed in the unpolarized PLE spectra (see also Figure 2c). However, the excitation energy at which the polarization degrees begin to increase is slightly different for particular lines.

Let us analyze in more detail the evolution of the helicity-resolved spectra as a function of the excitation energy for each PL line. The circular polarization degree of the neutral exciton X_A_ amounts to about 20%, when the laser energy is in resonance with the 3s state of the A exciton, and becomes weakly smaller with decreasing excitation energy. It reaches a minimum value of ~15% at an excitation energy equal to E_ex_ = 1.8631 eV. Here, the laser excitation energy and both ZO (hBN) + A′_1_(WSe_2_) and ZO (hBN) phonon modes are out of resonance with any optical transition of the WSe_2_ monolayer. When E_ex_ is further decreased, the polarization degree of X_A_ increases and saturates at an almost constant value equal to ~30%, when the complex Raman scattering mode ZO (hBN) + A′_1_(WSe_2_) crosses the XX^0^ energy.

The circular polarization degree of the neutral biexciton XX^0^ is about 40% at resonant 3s A exciton excitation, decreases weakly with decreasing excitation energy and reaches a minimum of ~25% when E_ex_ = 1.8631 eV. Interestingly, at this excitation energy the Raman mode ZO (hBN) + A′_1_(WSe_2_) is more intense than the emissions of all excitonic complexes. Although the combined ZO (hBN) + A′_1_(WSe_2_) phonon mode is close to the X_A_(1s) resonance, both lines are still well-resolved in the emission spectrum. A further reduction in the excitation energy yields a gradual increase in the polarization degree of XX^0^. It attains a broad maximum at ~60%, for energies between 1.8370 eV and 1.8249 eV. At an excitation energy of 1.8370 eV the ZO (hBN) + A′_1_(WSe_2_) phonon mode is located at the low-energy wing of the X_A_ emission; nevertheless, it can be resolved in the emission spectra. At lower excitation energy of E_ex_ = 1.8249 eV the combined phonon mode merges with XX^0^ and is not distinguished in the spectra.

Let us now analyze the excitation energy evolution of the circular polarization degree of the trion lines T_T_ and T_S_, as presented in Figure 4b. A subsequent decrease and increase in their polarization degrees with reducing excitation energy is observed similar to that of X_A_ and XX^0^. Additionally, for the trions, an intriguing variation of their relative polarization degree at particular excitation energies is revealed. At all excitation energies the polarization degrees of the triplet and singlet trion emissions are almost the same, except (i) for the laser excitation energies E_ex_ = 1.8567 eV and E_ex_ = 1.8475 eV, when the polarization degree of the triplet trion exceeds about 1.5 times those of the singlet trion, and (ii) for the excitation with energy E_ex_ = 1.8117 eV, when their polarization degree ratio is equal to about two. The clear difference in the polarization degrees of the triplet and singlet trions has been observed in previous reports [15,54,55] concerning WS_2_ monolayers (with the same ordering of spin-split conduction and valence band as in WSe_2_ monolayers) and has been attributed to their different dephasing rates. According to ref. [54], due to exchange interaction an electron–hole pair forming the triplet trion at one valley is scattered to the opposite valley and forms the singlet trion; thus, the triplet trion feeds the singlet trion. The efficient scattering of electron–hole pairs between opposite valleys needs a contribution of phonons with appropriate energies and momenta. This scattering process is more efficient at a double resonance when the ingoing and outgoing photons are in resonance with the singlet and triplet trions. This double resonance is achieved at particular excitation energies which manifests itself in a variation of the triplet-singlet polarization degree ratio. These particular phonons can originate from both the WSe_2_ monolayer and hBN as well as the Si substrate. The comprehensive understanding of the nature of these variations calls for further experimental and theoretical studies.

The circular polarization degree of the negatively charged biexciton XX^−^ is equal to ~30%, when the laser excitation is in resonance with the X_A_(3s), and exhibits a strong decrease with decreasing excitation energy reaching a minimum value of about 5% at E_ex_ = 1.8567 eV. The minimum polarization degrees of the XX^−^, X_A_ and XX^0^ emissions are detected at the same excitation energy. Further reducing the excitation energy results in a rapid growth of P of XX^−^, in a saturation of ~20% for E_ex_ ranging between 1.8498 eV and 1.8370 eV, and in a gradual decrease below 10% at E_ex_ = 1.8117 eV.

In Figure 4d, the circular polarization degrees of both Raman modes, namely the single ZO (hBN) and combined ZO (hBN) + A′_1_ (WSe_2_) mode, are plotted at excitation energies at which they do not merge with PL lines. As seen in Figure 4d, the ZO (hBN) mode is almost completely polarized at high excitation energies. For low E_ex_, it gradually loses its circular polarization down to about *p* = 50%. The polarization degree of the ZO (hBN) + A′_1_ (WSe_2_) mode remains at values between 40–50%. 

**Figure 5:**
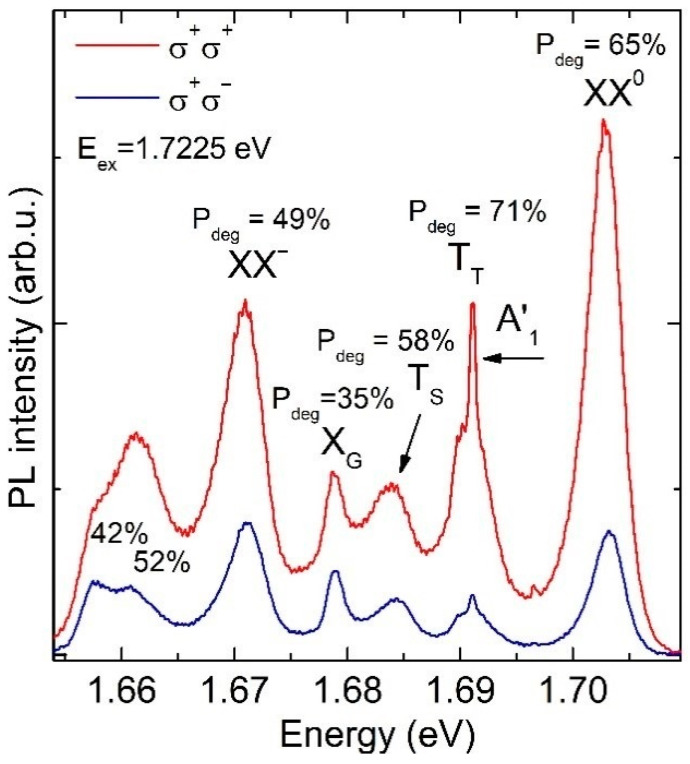
Helicity-resolved PL spectra recorded in two circular polarization configurations of exciting and emitted light: Co-polarized, σ^+^/σ^+^ (red line), and cross-polarized, σ^+^/σ^−^ (blue line). The laser light is in resonance with the X_A_ transition at 1.7225 eV. The origin and circular polarization of the emission of all lines are marked. The black arrow marks the position of the A′_1_ (WSe_2_) Raman mode.

Additionally, we performed polarization-resolved PL measurements with an excitation energy equal to the X_A_(1s) resonance. The spectra recorded in the co-polarized, σ^+^/σ^+^ (red line), or cross-polarized, σ^+^/σ^−^ (blue line), configuration are plotted in Figure 5. As seen from the comparison of the polarization degrees presented in Figure 3 and Figure 5, a high optical orientation of all lines (it is the circular polarization of the outgoing light with respect to the circular polarization of the excitation light) is also obtained for ingoing photons being in resonance with the 1s A exciton and thus due to interlayer electron–phonon coupling. Consequently, the optical orientation of all PL lines may be controlled by selective excitation.

## 4. Conclusions

We investigate the interlayer electron–phonon coupling in a van der Waals heterostructure composed of a WSe_2_ monolayer encapsulated in high-quality hBN crystals using reflectivity contrast and photoluminescence excitation experiments at *T* = 7 K. The intensity of the emission from the WSe_2_ monolayer is strongly increased due to a double resonance, where the laser excitation is in resonance with the 2s A exciton in WSe_2_ and the energy of the combined phonon mode ZO (hBN) + A′_1_(WSe_2_) is equal to the energy separation between the 2s and 1s exciton states. Accordingly, the outgoing photon is in resonance with the 1s A exciton in WSe_2_. We also find a remarkable impact of the interlayer electron–phonon coupling on the preservation of the exciting light helicity in the emission of the neutral and charged excitons and biexcitons. The highest value of the circular polarization degree of up to 60% is detected for the emission of the neutral biexciton and the negative triplet trion. The maximum polarization degree of the neutral A exciton was instead about two times smaller. Furthermore, an enhancement in the emission of the neutral biexciton XX^0^ at the double resonance condition is demonstrated.

## Data Availability

Data available on request due to restrictions eg privacy or ethical. The data presented in this study are available on request from the corresponding author.
